# Learning Experience of Chinese Nursing Students during Clinical Practicum: A Descriptive Qualitative Study

**DOI:** 10.3390/nursrep11020046

**Published:** 2021-06-21

**Authors:** (Anson) Chui-Yan Tang

**Affiliations:** School of Nursing, Tung Wah College, 31 Wylie Road, Homantin, Kowloon, Hong Kong, China; ansontang@twc.edu.hk; Tel.: +852-3468-6832

**Keywords:** nursing education, clinical learning, baccalaureate programme, learning experience, learning environment

## Abstract

The change in clinical environment can have a significant impact on nursing students’ clinical learning and as a consequence, to their competency. Students’ learning experiences could provide important insights for improving the existing approach towards clinical education. This descriptive qualitative study aimed to explore nursing students’ clinical learning experience. Focus group interviews were conducted with 20 final year nursing students studying a bachelor nursing programme at a self-financing tertiary institution in Hong Kong. Thematic analysis was conducted. 16 female and four male students were recruited. Six themes were identified: Anxiety towards clinical practicum, expectations of roles and responsibilities in practicum, ward environment, adequacy of support, learning attitude, and practicum arrangement. The findings suggest that nursing students are more discontented with their clinical training than before. Nursing faculty must look for possible ways to improve the clinical learning environment.

## 1. Introduction 

Clinical practice is a crucial component in nursing education. It enables students to transfer theoretical knowledge and skills learnt in school into real clinical situations [1,2,3]. The clinical component of pre-registration nursing training occupies nearly half of the time the students need to spend in the whole course of training [3,4]. The nursing faculty should therefore be sensitive to changes in clinical situations that will affect how well the training promotes constructive learning. Although many studies have reported facilitators of and barriers to positive learning in clinical education, evidence persistently indicates that students are dissatisfied with their clinical training. This dissatisfaction will almost certainly continue or even worsen in coming decades as global changes put increasing pressure on healthcare services including, if not particularly with regard to, manpower. The World Health Organization (2013) [5] has projected that about 40% of nurses may leave the profession in the coming 10 years. When the demand for nursing manpower outweighs the supply, clinical supervision becomes an extra burden on already challenged frontline nurses. It is reasonable to expect that the quality of learning will be significantly compromised in such clinical situations. 

The quality of a clinical learning environment is a determinant of the quality of the learning experience and learning outcomes [6,7,8]. The learning experiences in turn affect a student nurse’s transition into a professional nurse and, later, the decision of whether to remain in the profession [9]. Beneficial clinical experiences can help ease the transition by readying students to assume the work to be done in an actual clinical situation [10]. In contrast, inadequate learning opportunities will reduce their confidence and increase their sense of being unprepared [11,12,13]. Previous studies largely concur that active participation and involvement in clinical activities, effective communication between schools and staff nurses, and sufficient guidance from staff nurses are significant predictors of positive learning experiences [14,15,16,17,18,19]. Positive staff nurse attitude and positive role modeling motivate students to learn [8]. A recent study conducted by Kaihlanen et al. (2018) [20] summarizes the essential clinical practicum elements that lead to successful transitions of students into professional nurses as being: adequate supporting resources, good quality of final clinical practicum, and opportunities to prepare for the transition. 

Regardless of all the known positive factors, there are indications that the ways clinical practicum are currently being taught are not as good as they could be. Students have continuously and consistently indicated that they feel all aspects of clinical practicum could be improved [21,22,23]. Studies have found that students experienced various degrees of anxiety at the beginning of and during the practicum [15,24]. Fear of making mistakes and being assigned too many responsibilities ranked the highest in the stressor list [19]. Also, discrepant practices between the school and the clinical sites, the varied quality of clinical supervision and the inability to clearly differentiate their professional roles from other nursing auxiliaries made students feel frustrated and inferior [8,15].

Significant changes in the healthcare environment in recent decades will probably worsen the quality of the clinical learning environment. These trends result from a co-occurrence of regional and national changes and events, such as: rapid urbanization as people move from rural to urban cities in developing countries; influx of refugees from war areas to developed countries; outbreaks of contagious diseases; and frequent violent natural disasters due to global climate change. These changes result in challenges that can certainly overwhelm the healthcare system of a country. Hong Kong, being a cosmopolitan city, is no exception. The nursing turnover rate increased from 4.7% in 2010 to 5.4% in 2016. While this is comparable to rates reported in other countries [25,26], it negatively impacts the quality of frontline healthcare services. The latest statistics from The Hong Kong Nurse Association (2017) [27] show that the nurse-to-patient ratio in Hong Kong in recent years reached an unprecedentedly high level, namely 1:10–12, and a figure that profoundly exceeds the international standard ratio of 1:4–6. Nursing shortage has become a chronic and universal problem as clinics, hospitals, and community centers struggle to meet demand. While a commensurate increase in nurses’ training is one of the most pragmatic solutions to overcome the shortage, more nursing students implies that frontline nurses need to assume more teaching duties on top of the overwhelming patient care. It is quite understandable that the quality of their clinical supervision will be compromised. 

Nonetheless, the impact of those mentioned challenges on the clinical learning environment has yet been fully revealed. Only a handful of studies have reported on the clinical learning experience of nursing students in Hong Kong and those studies were conducted at least 10 years ago when the clinical situation in Hong Kong was significantly different than it is now. Lee and French (1997) [28] conducted a qualitative study to explore the learning experience of nursing students during clinical practicum. It found that the clinical learning environment did not favor effective learning. The staff nurses believed that students were there to carry out the routine care, instead of to learn. A quantitative study also reported that the clinical situation in Hong Kong was not supportive enough, and that students’ perceptions of the actual and ideal clinical learning environment differed greatly [21]. There is no current information on the actual impact of the changed healthcare environment on students’ learning over clinical practicum; as a result, the nursing faculty have no ways to understand how the quality of clinical learning has changed, if in fact it has. 

Therefore, this study aimed to explore and document the current clinical learning experience of Chinese nursing students in Hong Kong. With this knowledge, nursing faculty can modulate existing teaching strategies and improve clinical practice.

## 2. Materials and Methods

### 2.1. Design and Setting

This was a descriptive qualitative study using focus group interviews as a data collection method to explore the learning experience of Chinese nursing students in clinical practicum in Hong Kong. Focus group refers to a group of selected individuals discussing a specific topic in an organized manner to collect their views and experiences in relation to the topic. It is one of the most common methods used in qualitative studies to generate a rich understanding of participants’ experiences and beliefs [29]. As it is aligned with the objective of this study, it was used here. Nursing students enrolled in Bachelor of Health Sciences (Hons) programme (BHS) at a self-financing tertiary institution in Hong Kong were the target population.

### 2.2. Participants

Eligible participants were nursing students who were current students of the BHS programme and had passed all clinical practicum components stipulated in the curriculum. Those who had failed any one of the practicum components were excluded from the study. Convenience sampling was used to invite participants via email and poster on campus, and recruitment proceeded until data saturation occurred [30]. 20 students fitting the recruitment criteria were eventually recruited.

### 2.3. Data Collection

The interviews were conducted at the self-financing tertiary institution. The students were arranged in four groups of five students each; five is considered an acceptable number of participants for a focus group [31]. Each group was interviewed by the facilitator who led the group discussion according to a prepared guide. Another research team member, acting as an observer, took notes on the interaction of the group to supplement the oral text and enable a fuller analysis [31,32]. An interview guide with seven open-ended questions was designed with reference to Lee and French (1997) [28] and Sharif and Masoumi (2005) [15] to stimulate discussion regarding clinical experience. The question were

1. What do you feel about clinical practicum?
2. How is your learning experience during clinical practicum?
3. Is there any condition or incident that makes you anxious and worried during clinical practicum?
4. How did you face those difficulties and anxiety?
5. What factors affect your learning experience?
6. How can clinical experience be improved?
7. Are there any other things related to clinical practicum you want to share?

The questions were arranged from general to specific with the first two questions intentionally set as icebreakers to help participants feel relaxed in the group situation. The sequence of the subsequent questions was decided based on the relative importance of those topics to the research objective [33]. Probing questions were asked to go into more detail and guide the conversation further, such as ‘Can you tell me of a situation you encountered that led you to have such feeling?’ and ‘How did this affect you?’. Each interview lasted 60–90 min and was video-taped to capture the facial expression and the interaction among participants during the interview to supplement oral text and enhance data analysis [31,34]. 

To ensure consistency of interpretation of the interview guide and obtain comments from team members, the principal investigator briefed all the facilitators and observers on how to conduct the interview. A pilot interview was conducted among team members to make sure they were consistently and correctly interpreting the guiding questions. The way of facilitating the interview was also observed and comments were made to ensure the facilitation was done correctly and consistently. 

### 2.4. Data Analysis

Thematic analysis was used to analyze the interview transcripts. The interviewers transcribed the interviews within 48 h after finishing the interviews to ensure familiarity of the data [35]. The observer counterchecked the transcriptions to assure they were accurate based on his observations, particularly about the non-verbal behaviors and interactions of the participants during the interviews. The transcripts were then validated for accuracy by soliciting comments from the participants. Data from each group were coded, categorized, and analyzed by the author in accordance with the six phases of Braun and Clarke (2006) [35]. Initial responses to the data were generated by reading the transcripts several times to become familiar with the content. Relevant meaningful sentences to a corresponding interview question were extracted and coded systematically. Various codes were compared to identify similarities and differences. A tentative thematic map was formulated by collating initial codes into potential themes and sub-themes. The initial thematic map was compared with the coded data extracts again to look for discrepant interpretations; this comparison facilitated further refinement of the themes and sub-themes. To validate the analysis, the results were returned to the participants to check for accuracy and congruence with their experience. 

### 2.5. Ethical Considerations

Ethics approval was sought from the school research committee of the nursing school of the self-financing tertiary institution. All the participants were well informed of the purpose and potential benefits and harm of the study. They were told that the whole interview would be video-taped to facilitate subsequent analysis. Their performance during the interview was independent of their academic performance. The participants were free to leave the interview at any time without penalty of any kind. Information sheets were issued and written consents were obtained before the commencement of interviews.

## 3. Results

All 20 participants were final year students of the BHS programme; most students were female (*n* = 14) and all were aged 23–25. All groups expressed similar views towards clinical practicum. From the analysis, six overarching themes emerged regarding their clinical experience; these were: anxiety towards clinical practicum, expectations of roles and responsibilities in practicum, ward environment, adequacy of support, learning attitude, and practicum arrangement. 

### 3.1. Anxiety towards Clinical Practicum

All focus groups expressed that they felt anxious before and during the clinical practicum. Sources of anxiety mainly came from the sense of incompetence, criticism from clinical mentors, sense of insecurity, and loss of freedom. One of the participants expressed this anxiety as follows:

*‘I lacked confidence to perform nursing procedures, which was new to me as I didn’t have experience of doing them when I first underwent the practicum.’*

Another participant said:

*‘I never work in hospital, I do not know what I should know and do…I felt I didn’t prepare well…’*

Criticism from clinical mentors was quite a significant source of anxiety to the students. One of participants said,

*‘I did have hard feeling as my mentors often told me that I didn’t do good enough when I was taking care of patients….it made me down.’*

Another participant said:

*‘I was afraid of being scolded by staff nurses because I wasn’t skillful and knowledgeable enough…’*

Sense of insecurity was reported as students needed to carry out the duties without close guidance. One of the participants explained that:

*‘I had to carry out some nursing care independently with limited guidance from the clinical mentor which made me nervous and fear of doing the tasks.’*

Another participant stated that:

*‘If we were coached by the school teacher, she would remind you and guide you what and how to do…the sense of security is higher.’*

Interestingly, students also felt a loss of freedom to do their work because they were constantly monitored by others. One of participants said:

*‘I was under supervision of the other staff nurses. I couldn’t carry out the work by myself without asking the permission from the nurses for even doing simple care like tube feeding.’*

### 3.2. Expectation of Roles and Responsibilities in Practicum 

This theme is defined as the discrepancy between what the students expected their duties to be and what their supervisors expected them to do. All participants said that they felt stress because of the high expectations from the staff nurses. One of the participants said:

*‘All staff nurses expected me to know all the knowledge and skills……they would expect me to have encountered all different procedures and cases…Final year students will be scolded by the nurses if they don’t.’*

Another participant stated that:

*‘Once the student doesn’t meet their standard, the staff will think that he/she is stupid and will be scolded by them……This impedes our active learning.’*

Participants voiced a feeling that they were always being treated like a health care assistant. They had to perform all the routine care. They felt that they lost learning opportunities a student should have. A participant expressed this discrepancy on expectations of roles and duties as follows:

*‘Students were being health care assistants instead of students … Students were supposed to learn things but not being counted as part of the manpower in a ward.’*

### 3.3. Ward Environment 

This theme includes ward culture, including clinical teaching atmosphere and ward practice, and staff workload. Most participants stressed that the clinical sites now were unfavorable to student learning. Participants seemed encountering staff nurses unwelcomed nursing students. One of the participants described this sense of alienation as follows:

*‘I had been a ward with poor teaching atmosphere. They weren’t willing to coach students or even didn’t welcome nursing students.’*

Another participant echoed:

*‘Some staff seldom coached and offered opportunities to students. They thought that students were transient guests in the ward...they were no bother to teach you…’*

Ward practices were different in different units and even in the same unit. There were no explicit guidelines as to how they should work. Having an informative orientation seemed a reasonable solution to help adaption. Participants said:

*‘I would get lost when I rotated to a new ward as the routine, setting, and practices are totally different...the staff had different practices, I don’t know which one I should follow…’*

*‘An informative orientation is very important to students. Some had comprehensive orientation, some just give us a note without explanation… we would easily get things wrongly done without a proper orientation……and scolded by nurses eventually.’*

The workload of frontline nursing staff gave them no extra time to teach. A participant said:

*‘Due to insufficient manpower, I was expected to be one of them……Basically, I had no way to learn but just finished all the routine work in the ward...’*

Another participant expressed concern as follows:

*‘The learning burdened a busy ward. I lacked time and opportunities to learn…… The nurses also lacked time and patience to coach me and answer my enquiries.’*

### 3.4. Adequacy of Support 

Sufficient support from clinical staff, peers and nursing faculty was of prime importance to students’ learning. The present mentorship system disappointed students and impeded, rather than facilitated, their learning. A participant explained disappointedly:

*‘A mentor was assigned to each student but they seldom met each other because different shifts were being assigned or he/she was on vacation leave. It is futile to have a mentor which can hardly meet. They just helped fill out the evaluation form. ‘*

Another participant described her experience as follows:

*‘I expected the mentor would set out learning objectives with me and taught me the techniques in case management. However, in the current low staffing situation, they didn’t…they never approached me… when I wanted to learn how to handle cases, they would ask you to do the routine work…’*

Another participant said the staffs’ attitude refrained them from going further to learn:

*‘Some staff thought that final year students should have known all the stuff. If you asked them, they would show unpleasant gesture…their attitude turned me down… I am more willing to ask them (nurses) question if they are nicer and look more approachable.’*

School support plays a vital role in the whole learning experience. Participants believed that the school should take measures to ensure students learn effectively in the practicum. School support includes the role of nursing faculty and preparatory work for clinical practicum. Participants agreed that the nursing faculty should visit students regularly to give both factual learning and psychological support. A participant said:

*‘Resource person (nursing faculty) came and ask me cases. I had to share cases and what I had done on the patients…they told me whether I did it right or not…sometimes, if I told them how the ward treated me badly, they would advise me how to overcome it, solve the problem…They should come more frequently…some came once a week, some once bi-weekly… ’*

Another participant said:

*‘There were insufficient visits and guidance from resource person in the final clinical practicum…… I wanted her to meet me at least once per week to teach me case management and discharge planning.’*

Participants largely believed that a practicum preparatory workshop could equip them better for the practicum; however, some believed that the content of a workshop could not reflect what they exactly did in the ward. A participant said:

*‘The (practicum preparatory) workshop is useful. It can brush up my basic skills…it also teaches me how to handle cases. It isn’t what I will do in the ward, I can’t handle cases in the ward…but it can give me a concept how the ward or patient care will be…’*

Outside school, classmates and nursing students from other institutions acted as a backup to their learning as well as outlet for their pressure. All participants concurred that peer support was vital to their learning. A participant commented that:

*‘The presence of other students could share the workload which lowered my stress level……It could also enhance the efficiency of our work, so we could spend more time to learn new stuff... Other students were willing to pick up your work……In my experience, adequate support from other students to share the workload can minimize the chance of being scolded.’*

### 3.5. Learning Attitude 

This theme refers to the appropriate attitude nursing students should have in order to increase learning opportunities. One of the participants stressed that:

*‘Students should be proactive enough to grasp their own learning opportunities……they should be courageous enough to approach the staff and request to take part in nursing care or observe the procedures...if the staff doesn’t know what they don’t know, they couldn’t help and coach you.’*

Another student opined:

*‘…I thought it is related to the attitude, Staff is more willing to teach you some new knowledge or allow you to participate in nursing care if you are helpful, cheerful and humble but not mean to others…if the staff and students get along well, it is easier to ask for new tasks or request them to supervise you for an unfamiliar task.’*

A majority of the students explained that they would not argue when facing criticism. A participant stated that:

*‘I had an experience of being condemned by a staff. I felt sad and innocent at that moment as I had done nothing wrong……I could just keep silent……The only thing I could do was to seek support from friends after the duty.’*

### 3.6. Practicum Arrangement

This theme refers to how clinical practicum is organized, including duration of each practicum block and duty pattern. All students recommended that each practicum block should be shorter. One of the participants said:

*‘The duration of the final nursing practicum which lasts for half a year is too long…… It affected my learning as I did’t have enough time to consolidate the things I learnt.’*

In regards to the duty pattern, students reported that they were not used to shift duty and that one rest day per week was not adequate. A participant explained that:

*‘Apart from clinical practice, we also need to put effort on other assignments while we only had one day-off each week……I was stressful because of lacking rest and personal time…’*

Another participant said:

*‘The shift duty pattern made me lose my social life…I feel depressed to get up so early that the sun still hasn’t risen to catch up with the morning duty. Sometimes, I didn’t’ sleep well between morning and night shift. I will take Piriton to help sleep.’*

## 4. Discussion

This study revealed that students’ dissatisfaction towards clinical learning persists and that they continue to experience anxiety in clinical practicum. Some reasons for anxiety, such as insufficient knowledge and skills, and fear of harming patients, correspond to those mentioned in previous studies [15,19,24]. Other factors, namely, sense of insecurity, loss of freedom, and criticism from mentors, are newly revealed. It seems contradictory that, on the one hand, students are afraid of losing freedom when doing things under supervision while, on the other hand, they feel insecure to do things with minimal guidance. Such dilemma might be explained by the intrinsic characteristics of young people in the 21st Century. The nursing students in this study were born in the late 1990s, the generation being labelled as Millennials. Millennials have grown up in an era of knowledge explosion and technology advancement. They can easily get access to the latest information through the internet, and the media they are exposed to cultivate in them a mindset of challenging traditions and authorities [36,37]. With the concerted effect of social influence and nurturing from schools, students are used to a learning atmosphere that enables open discussion and argument. In contrast, the learning model adopted in clinical education is still largely a prescriptive apprenticeship approach [7]. This approach emphasizes rote learning and obedience. Students are part of the manpower of the hospital and are subordinate to senior staff nurses. The discrepant learning environments between school and clinical sites as well as the mismatch of the expected behaviors of a nursing student and the intrinsic characteristics of Millennial students may therefore make them uneasy when facing criticism and needing to strictly follow instructions. 

The dissatisfaction with clinical learning seems to be intensifying as compared to a few decades ago. In this study, all participants expressed the belief that they had had unfavorable clinical experience; this differs from findings in other studies [15,21,38]. Sharif and Masouni (2005) [15] reported that half of the participants came across senior nurses willing to coach students. Chan and Ip (2007) [21] found that students rated mentorship in actual practice higher than expectations. Warne et al. (2010) [38] launched a national study to explore nursing students’ experiences in clinical learning environments in nine European countries. Only 14% of the respondents reported discontent with their clinical practicum. The intensified unfavorable clinical learning experience implies that the clinical learning environment nowadays is deteriorating and becomes ever more challenging. A challenging clinical learning environment has two components—context of learning (including curriculum design and delivery) and relationships with nursing faculty and staff nurses (including availability of role models) [17]. Negative relationship with staff nurses, unsupportive practice cultures, lack of role models and inappropriate practicum arrangement may jeopardize the whole learning experience [8,17]. Unfortunately, the current clinical learning environment seems to possess all of these undesirable elements. 

Mismatched duty rosters, unsupportive staff attitude and incompatible expectations of student nurses’ roles and responsibilities result in communication breakdown, which subsequently compromises the nurse–student relationship. Sincerity and approachability on the part of the staff nurses can alleviate students’ anxiety and make students feel that they are included and that it is safe to ask questions and explores practices so to enhance the learning [7,17,38]. In contrast, lack of respect and aloofness on the part of staff nurses can only put pressure on students, undermine their confidence, and impede learning [18]. Given the chronic shortage of nurses and the conventional prescriptive training modality, it is understandable that clinical staff would view student nurses as a good, even welcome, source of manpower. It seems a desirable approach for students to learn as they are being included in the team, sharing nursing tasks, so they can gain hands-on experience [7]. The problem is that, in practice, the involvement in the team is confined to routine care; it seldom includes other nursing activities, which a qualified nurse is expected to do. 

Lack of role model is another significant issue. Due to the high turnover rate and massive recruitment of newly graduate nurses, the proportion of relatively junior nurses in hospitals is increasing. They have not yet built-up sufficient experience in clinical area and so it is reasonable to think that they may not be able to supervise students properly [17,39]. Although the school usually provides briefing sessions for staff nurses designated as clinical mentors, the fact is that these mentors might not have the same shift as the assigned students. As a result, students might either have no mentor to supervise or be supervised by staff nurses who did not receive proper training in clinical mentoring. It is thus no surprise to learn that students demand more from their nursing faculty as reflected from the findings. The dissatisfaction also affects their perception of the practicum arrangement. Warne et al. (2010) [38] found that students with seven weeks or more of placement rated the learning atmosphere in the ward and mentorship relationship significantly higher than those with less than seven weeks of placement. However, in this study, this was not the case. The findings in this study suggest that students preferred a practicum with shorter duration instead. This might reflect their dissatisfaction with the learning. 

There is no single recipe to manage this undesirable situation. Nursing faculty should be fully aware of all these unfavorable learning elements in the present clinical learning environment, and they must look for appropriate and effective measures to improve them. The participants mentioned that support from nursing faculty is as important as support from staff nurses. Nursing faculty may need to enhance and strengthen their facilitative role in clinical education in order to ensure that students obtain sufficient opportunities to develop and demonstrate the required nursing competences, such as psychomotor skills, clinical reasoning, and that they receive regular feedback for reflection and improvement [20]. Furthermore, tailor-made and informative orientations should be offered to staff nurses. Such orientation could help facilitate them in understanding the requirements of the practicum, the capabilities of the nursing students, and the expected roles and responsibilities of the students in clinical practicum. This should help narrow the expectation gap between staff nurses and students, and thus enhance the mentorship. The nursing faculty should understand the unique characteristics of Millennial students and initiate measures to nurture in them the appropriate interpersonal skills and learning attitude during the clinical practicum. 

This study provides latest evidence regarding how nursing students feel and what they are experiencing over clinical practicum in a recent clinical environment. It provides nursing faculty with a new insight on how to strengthen the support and preparation before and during the clinical practicum. There are two limitations in this study that may affect trustworthiness of the findings. First, the subject recruitment was taken from only one tertiary institution in Hong Kong. The programme structure and support provided to students may vary among other nursing programmes in Hong Kong. Second, the participants were all final year baccalaureate nursing students who have comparatively more clinical experience that their junior counterparts. Their learning experience in a clinical environment may not be able to fully reflect the feeling and experience of junior students. 

## 5. Conclusions

According to the results of this study, current nursing students are even more dissatisfied with their clinical learning than the past ones. Students were anxious toward clinical practice as they felt themselves not yet ready to work in the clinical sites; they were afraid of losing freedom and being criticized by staff nurses. Aspects of clinical practicum that need improvement include: the teaching atmosphere in the clinical sites, adequacy of support from staff nurses, nursing faculty and peers, practicum arrangement; expectation of roles and responsibilities in practicum; and learning attitude. Nursing faculty should give attention to these areas in order to improve the learning experience of the nursing students. The benefits of such improvements will be better trained, more satisfied and dedicated nurses and a better healthcare system for the community. 

## Data Availability

Not applicable.
